# Different Types of Survey-Based Environmental Representations: Egocentric vs. Allocentric Cognitive Maps

**DOI:** 10.3390/brainsci13050834

**Published:** 2023-05-22

**Authors:** Maria Kozhevnikov, Jyotika Puri

**Affiliations:** 1 Department of Psychology, National University of Singapore, 9 Arts Link, Singapore 117572, Singapore; jyotika.puri@duke-nus.edu.sg; 2 Martinos Center for Biomedical Imaging, Harvard Medical School Department of Radiology, 149 Thirteenth Street, Charlestown, MA 02129, USA

**Keywords:** survey-based environmental representations, egocentric cognitive map, path integration, egocentric landmark processing, map-based navigation

## Abstract

The goal of the current study was to show the existence of distinct types of survey-based environmental representations, egocentric and allocentric, and provide experimental evidence that they are formed by different types of navigational strategies, path integration and map-based navigation, respectively. After traversing an unfamiliar route, participants were either disoriented and asked to point to non-visible landmarks encountered on the route (Experiment 1) or presented with a secondary spatial working memory task while determining the spatial locations of objects on the route (Experiment 2). The results demonstrate a double dissociation between the navigational strategies underlying the formation of allocentric and egocentric survey-based representation. Specifically, only the individuals who generated egocentric survey-based representations of the route were affected by disorientation, suggesting they relied primarily on a path integration strategy combined with landmark/scene processing at each route segment. In contrast, only allocentric-survey mappers were affected by the secondary spatial working memory task, suggesting their use of map-based navigation. This research is the first to show that path integration, in conjunction with egocentric landmark processing, is a distinct standalone navigational strategy underpinning the formation of a unique type of environmental representation—the egocentric survey-based representation.

## 1. Introduction

The intriguing topic of how individuals generate their representations of space and what kind of navigational strategies lead to forming these representations has spurred a multidisciplinary pool of studies [1], ranging from neurobiology to behavioral and cognitive psychology. Motivated by the discovery of place cells, O’Keefe and Dostrovsky [2] proposed that it is the hippocampus that supports spatial processing and is involved in the generation of a cognitive map (i.e., an abstract “map-like” mental representation of one’s physical environment). Follow-up discoveries of other cells in the hippocampal region (e.g., grid cells, head direction cells, boundary cells) have further supported the critical role of the hippocampus in cognitive map formation. The cognitive maps (also referred to as *survey knowledge* of the spatial layout of known places) have been suggested to be *allocentric* (i.e., positions of objects, landmarks, and places and their interrelationships are encoded in relation to a framework external to the navigator [3] and have a metric Euclidean structure [4,5,6]). Furthermore, such allocentric cognitive maps have been proposed to encode “fragments” of the environment holistically and cohesively in a common coordinate system, thus affording long-term memory for the spatial relationships between places [7,8].

Earlier studies on spatial navigation [9,10] suggested that the formation of cognitive maps is the result of *allocentric navigation*. Allocentric navigation (also termed *map-based navigation* or *piloting*) refers to the process of selecting and maintaining a path from one place to another using spatial relations between allothetic cues (i.e., externally generated signals, such as landmarks or features of a boundary) [11]. *Procedural* or *route-based navigation* [12], during which a traveler memorizes a temporal sequence of associations coupling salient landmarks or scenes with corresponding motor responses (e.g., after reaching the coffee shop, turn left), was considered an alternative strategy to allocentric navigation. In contrast to allocentric navigation, procedural navigation does not support cognitive map formation; it is limited to navigation between familiar places and results in less flexible wayfinding behavior (e.g., difficulties in finding shortcuts or novel routes). Subsequent studies, however, have shown the critical importance of another navigational strategy, such as path integration, in wayfinding performance. During path integration, a traveler performs continuous or discrete updating of their current position and orientation relative to the travel origin [3,13,14].

In continuous, moment-to-moment updating, the traveler is continually estimating their current position and orientation based on travel velocity and the prior estimate of position and orientation. In configural updating, the traveler maintains a representation of the traveled path and often updates the estimate of the current position and orientation based on the estimated starting location and the stored representation of the path [13]. According to the model of path integration adopted in this paper, egocentric self-to-object distances and bearings are coded independently of the global layout of the environment. Yet, according to other models of path integration, the egocentric representations of surrounding locations formed during path integration are subsequently integrated into a cohesive allocentric cognitive map [15,16,17,18,19,20]. In contrast to allocentric navigation, which relies on spatial memory of the landmark locations and the allocentric spatial relationships they specify, path integration involves tracking transient egocentric (self-to-object) representations of surrounding locations based on the idiothetic signals, i.e., generated by self-motion, such as vestibular (inertial) and proprioceptive cues, optic or acoustic flow, and efferent copies of movement commands [6,15,21,22,23,24].

Consistent with the views above, numerous studies on individual differences in spatial navigation have primarily distinguished between the corresponding preferences (referred to as “navigational styles”) for either allocentric/map-based or procedural/route-based navigation, often assessed via self-report questionnaires [25,26,27,28,29]. In these studies, the procedural/route-based navigation style has been related to the formation of procedural/route-based representations, reflecting the sequential linking of segmented paths without preserving the spatial layout of the route. In contrast, allocentric/map-based navigational strategies have been related to allocentric survey-based representations formed by the spatial integration of landmark configurations. Individual differences in path integration abilities, however, have received much less attention.

However, there has been accumulating evidence that there might be another type of cognitive map, which is orientation-specific and encodes the route segments with corresponding landmarks from the egocentric rather than the allocentric perspective [30,31,32,33,34]. These maps can be traced back to the differences in the ancient and medieval cartography of European versus Islamic, Chinese, and Southeast Asian cultures [35,36]. In contrast to modern European maps, which are schematic representations of an environment, accurately depicting locations and distances between the landmarks and portraying the environment using cardinal directions (orientation to the north) in a top-down (allocentric) manner, older and even some modern Islamic, East Asian, and Southeast Asian maps can be described as “coordinate-free”, as they include several chunks of landmarks of varying orientation, representing different geographical regions, portrayed from the first-person perspective, without being embedded into any coordinate system.

The experimental evidence that large space memories might encode information in an orientation-specific rather than allocentric manner comes from several studies (e.g., [37,38,39]) where the participants were guided on a path (learning phase) and then asked to point to different, not directly visible locations. In the experiment by Waller, Montello, et al. [39], participants in all conditions, including “direct walk,” during which they were able to move through an environment and update their position and orientation in space, demonstrated a significant alignment effect (i.e., better performance on pointing to locations while imagining themselves facing the same directions as during the learning phase). In the study by Wang and Spelke [37], after learning an environment, participants performed significantly worse while pointing to non-visible targets in the disorientation condition. In particular, the configuration error—which indicates the accuracy of the localization of each target in relation to the others—significantly increased after disorientation, casting doubt on the enduring “observer-free” cognitive maps. In another study, Chrastil and Warren [31] proposed that the majority of participants, after freely exploring a novel environment, acquired “labeled graph” knowledge of the environment (i.e., an environmental representation that incorporates local metric information about distances between known places and/or angles between known paths), but not allocentric Euclidian cognitive maps. Recently, Warren et al. [40] expanded the concept of “labeled graphs” to “cognitive graphs”, emphasizing the findings that these representations are not embedded in a global coordinate system. The cognitive graphs have been proposed to be superior to procedural environmental representations as they afford to find shortcuts (e.g., shortcuts are generated on the fly by vector addition along the shortest path through the graph) but inferior to allocentric cognitive maps as they are coarser, contain biases, and are not globally consistent [34,40]. Warren [34] speculated that cognitive graphs are likely to be generated by path integration, as this navigation strategy is better suited for piecewise measurements of path lengths and turn angles than for forming a cohesive allocentric map (see also [41]).

Rather than focusing on whether survey-based environmental representations are orientation-specific or orientation-free, several studies [30,33] suggested that both might exist and that individual differences in visual-spatial abilities or sensorimotor capacities affect the type of environmental representations people form. In one of the studies [30], participants were asked to draw sketch maps of the path after exploring an unfamiliar route in a two-story building. In addition to the sketch maps that represented route knowledge or allocentric survey-based sketch maps that illustrated the holistic configuration of the route from a top-down perspective, Blajenkova et al. [30] identified a unique type of sketch map (egocentric survey-based sketch maps) that represented a combination of several route segments and topographical features, each presented from a first-person perspective. Although these sketch maps were similar to some of the procedural/route sketch maps with respect to the adoption of the first-person perspective, they accurately depicted the spatial layout of the route. While there was no difference between the allocentric- and egocentric-survey mappers, both were significantly higher in visual-spatial ability than the procedural mappers. In another experimental study, Zhong and Kozhevnikov [33] reported the existence of similar egocentric survey-based representations encoded after real-world route learning, which included several segments of the route drawn from the orientation consistent with the facing direction of the subjects while they were traversing the route. As egocentric-survey mappers scored the highest on the path integration scale of the self-reported Navigational Strategy Questionnaire (NSQ) compared to procedural and allocentric-survey mappers, Zhong and Kozhevnikov [33] suggested that this group of mappers might have acquired orientation-specific memories of the route as a result of path integration.

The experimental evidence that egocentric survey-based representations are formed by path integration, however, is still lacking. It also remains unclear whether the egocentric survey-based representations are “inferior” to allocentric representations (i.e., they are formed by people with less visual-spatial or sensorimotor capacities and might become orientation-free with repeated exposure to an environment) or they represent a qualitatively different type of survey-based knowledge that is formed by a distinct navigation process. The goal of the current study was to provide experimental evidence that egocentric survey-based representations are developed as a result of path integration, occurring within an egocentric framework, in conjunction with the encoding of landmarks and scenes in an orientation-specific manner. This is in contrast to allocentric survey-based representations, which we suggest are formed as a result of allocentric navigation by encoding landmark positions in a view-independent manner and integrating them in spatial working memory into a coherent allocentric map. Furthermore, taking into account considerable individual differences in forming environmental representations, we predict that, depending on their visual-spatial abilities and path integration capacities, some individuals may generate egocentric survey-based environmental representations, while others may develop allocentric survey-based representations or rely on procedural strategies.

In Experiment 1, we used the disorientation paradigm to compare the accuracy of the spatial representations of different navigators before and after disorientation. Research suggests that disorientation, such as spinning the subject around for a sufficient time, disturbs a sense of direction and orientation [37,42] and therefore disturbs the generation of egocentric representations. The disorientation paradigm has also been used in studies of spatial learning, which attempted to minimize the influence of path integration during spatial learning tasks by forcing the subjects to rely on allothetic instead of idiothetic cues (e.g., [43,44]). Thus, if egocentric-survey mappers rely on path integration strategies to construct their cognitive maps, their ability to determine the spatial locations of objects and their self-position would be significantly influenced by disorientation. On the other hand, individuals who form allocentric survey-based representations should not be influenced by disorientation, as they still have their enduring allocentric representations of an environment [8,45,46].

In Experiment 2, by introducing a secondary spatial working memory task, we examined the extent to which different types of navigators rely on spatial working memory while determining the spatial locations of objects in the environment. The formation of an allocentric survey-based representation heavily relies on memory for the spatial parameters (routes taken, landmark positions, environmental boundaries) and their integration in spatial working memory into a global allocentric map. In contrast, during path integration, travelers update their current position and orientation automatically or by simple vector addition, which requires significantly less reliance on spatial working memory than allocentric navigation. Therefore, if allocentric-survey mappers rely on allocentric/map-based navigation, their performance on tasks requiring determining spatial locations of landmarks relative to each other would be significantly more impaired than egocentric-survey mappers when they had to complete a secondary spatial working memory task.

## 2. Experiment 1

### 2.1. Methods

#### 2.1.1. Participants

Seventy-one participants (33 males), 20 to 26 years old (M age = 22.35 y.o., SD = 1.67), were recruited through the Psychology department’s research participation program at the National University of Singapore or an online advertisement. A priori power analysis was conducted using the G*Power 3 statistical software package [47], with an effect size set to 0.30, an alpha level of 0.05, a correlation between repeated measures of 0.5, and a statistical power of 80%, yielding a minimum sample size of 48 participants. To ensure a balanced number of participants in each group of navigators (procedural, allocentric survey-based, and egocentric survey-based), the self-reported Navigational Strategy Questionnaire (NSQ) assessing individual differences in different navigational strategies was administered to the participants prior to the experiment. The participants were compensated either via course credits or monetary reimbursement, respectively. All participants were recruited based on the prerequisite of being unfamiliar with the premises around the NUS School of Engineering, where the experiment was taking place.

#### 2.1.2. Route Traversal

**Figure 1:**
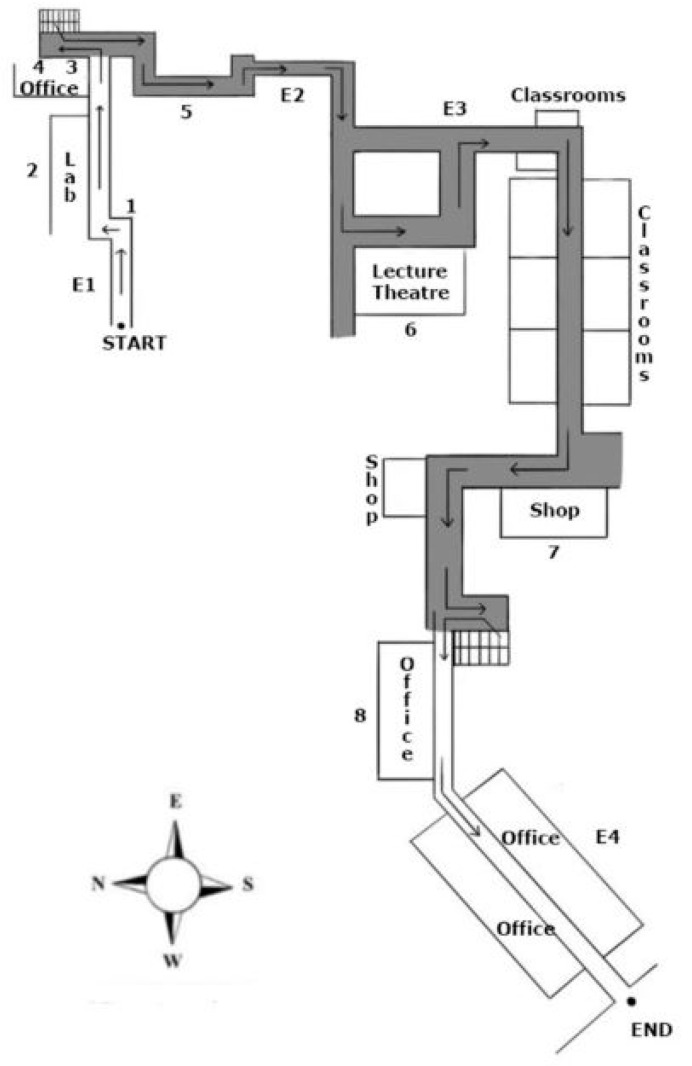
Floor plan of the route at the Faculty of Engineering (FoE) at the National University of Singapore (NUS). The black dot near “START” represents the start of the route, and the black dot near “END” represents the end of the route. Arrowheads represent the direction along the leg of each segment that the participant traverses. The numbers from 1 to 8 indicate the eight landmarks pointed out to each participant while walking the route. In sequence, the landmarks are as follows: (1) Brown Bench, (2) Systems Lab, (3) IBM Office, (4) Sembcorp Lab, (5) Open-air walkway, (6) Lecture Theatre, (7) Cheers convenience store, and (8) Staff Lounge.

Each participant was led by the experimenter individually on a real-world route that spanned four buildings at the Faculty of Engineering at the National University of Singapore (Figure 1). Participants were asked to memorize the route, including the starting point and eight landmarks the experimenter pointed out along the route, using any strategy they deemed appropriate. These landmarks were selected based on their inherent salience (e.g., Brown Bench, Cheers convenience store). They were either located at turning points on the route, like landmarks 1 and 7, or large and imposing structures, such as landmark 5. The route began at Engineering Building E1, where participants were led straight down a covered path toward the first landmark. After a quick left, they walked through a set of glass doors into an enclosed corridor and passed landmark 2. They walked down the corridor, turned left past landmark 3, down a set of stairs, and out to face landmark 4. Turning left at landmark 4, participants were led down to the fifth landmark, to an adjacent Engineering Building E2, and then down another linkway to Engineering Building E3, which houses the sixth landmark. Going past landmark 6, they were led to the next turning point, where they turned right to walk down an enclosed, brightly lit corridor with landmark 7 at the end. Finally, they walked down a connected corridor, took a flight of stairs up a level, and turned into a corridor with their final landmark 8 to their right. After that, they walked that corridor until the end point, marked in Figure 1 as “END”.

There were tables and chairs placed in the space where the route ended. The participants were seated on a chair facing southeast at the endpoint, so the landmarks were spread out around them. None of these landmarks were visible to the participants from the endpoint. The starting point was also hidden from view at the end point. Overall, the route was planned to make participants travel through multiple floors across multiple buildings through the Faculty of Engineering.

### 2.2. Tasks and Materials

#### 2.2.1. Triangle Completion Task

This task was adapted from the triangle completion task used in previous studies to assess the abilities of participants to update their location and orientation using path integration in the absence of visual cues [13,48,49,50,51]. The task was conducted in an indoor, vacant 6 m × 6 m space. Participants were blindfolded during the entire task duration, except during the dissemination of instructions and the practice trials. The experimenter led blindfolded participants on a path with two legs per trial. The length of each leg (2 m, 4 m, and 6 m) and turn angle (60°, 90°, and 120°) varied from trial to trial. At the endpoint of the second leg, participants were instructed to point back with a pointer from the endpoint to the origin. The responded return bearing was recorded based on a mobile compass application. The heading error, reflecting the difference between the responded return bearing (actual pointing direction) and the true return bearing, was computed. After completing each trial, participants were led back to the origin; the order of trials was counterbalanced across subjects. Each participant underwent a total of seven trials, the sequence of which was counterbalanced across participants. Based on the results of previous research [50], 2 out of 9 trials were dropped from this study as they did not provide enough variation in participants’ scores (i.e., most participants responded correctly on these trials). Heading errors for all seven trials were averaged to obtain a single value per participant. The duration of the task was approximately 10 min.

#### 2.2.2. Sketch Map Task

The sketch map task aimed to assess different types of mental and environmental representations formed by the participants. The participants were given the following instructions: “Please sketch out a map of the route you have just traversed from the start to the end. Please include as many route and topographical features as you possibly can. Make sure your lines are clearly drawn, and your landmarks are properly labeled. Please illustrate your map to the best of your abilities”, followed by blank sheets of A4 sized papers (21 cm × 29.7 cm), pencils, pens, and rulers to draw out their route. The participants were given 10 min for drawing and extra time when required. On average, each participant took between 10 and 15 min to draw out their map. Participants were then asked about their recall process to clarify any cues or strategies they used while remembering and drawing the map. Their responses to these questions were audio recorded. These responses were used to facilitate the classification of sketch maps in cases of disagreement among judges regarding their category.

#### 2.2.3. Route-Pointing Direction Task (R-PDT)

The R-PDT was administered to the participants at the end of the route. The task required participants to indicate the direction of eight landmarks, pointed out to participants while they were traversing the route, relative to their final heading direction. Specifically, this task aimed to assess participants’ performance at retrieving self-to-object relations updated during route traversal. The task is considered one of the classical assessments of large-scale environmental knowledge and, in particular, spatial updating, which requires participants to make estimates of the directions of non-visible landmarks situated in the environment surrounding their current position [51].

**Figure 2:**
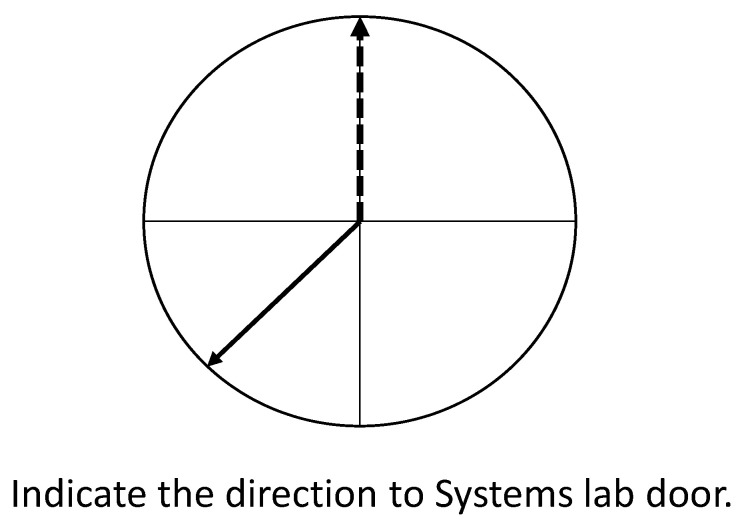
A sample trial of the route pointing direction task (R-PDT) wherein the participant has marked the heading of the landmark in question correctly (indicated with a solid black line) relative to their facing direction at the end of the route (indicated with a black dotted line).

At the end of the route, all participants were instructed to sit facing the same heading direction they faced while completing the route (southeast), from which none of the eight landmarks were visible. Then, they were given a pen and a paper worksheet containing four circles, each divided into four quadrants with the name of one of the landmarks and asked to indicate the direction to this landmark from their heading direction (Figure 2).

Participants were asked to indicate the direction of each of these landmarks relative to their current location and heading by drawing an arrow pointing in the direction they thought each landmark was located. After completing the first four trials, participants were required to stand up from the seat and spin around with their eyes closed for about 45 s to induce disorientation. After the disorientation, the participants were asked to return to the same seat, facing the same direction, and rest for another 20 s. After informing the experimenter that they were no longer experiencing any dizziness or motion sickness, they were given a second worksheet containing four circles with the names of the other four landmarks indicated on them. Similar to the first part of the task, the participants were asked to draw an arrow, indicating the direction to each of these landmarks relative to their current location and heading. The absolute deviations from the correct angles were computed for each of the eight trials. The participant’s mean absolute error (mean absolute deviation) was derived for the four trials before the disorientation (Time 1) and the four trials after the disorientation (Time 2).

To ensure equal difficulty in the first and second parts of the route-pointing tasks, we first matched all the landmarks to be included in the first and second sets of the R-PDT (before and after disorientation, respectively) on their imagined heading (i.e., the degree of mental self-reorientation required from the participants at the end of the route to imagine themselves facing the original perspective at which they encountered a particular landmark). The average imagined heading for the landmarks in the first and second sets of landmarks was 68° and 90°, respectively. Second, we conducted a pilot study with 11 participants, who were asked to point to all 8 landmarks without disorientation during the R-PDT task. Based on the average absolute deviation of participants from the correct pointing direction for each landmark, the landmarks to be included in the first and second parts of the study (before and after disorientation, respectively) were matched for their difficulty (see Table 1). There was no difference in participants’ overall performance on the first and second parts of the task; the paired sample *t*-test (two tailed) was not significant, *t*(10) = 0.1, *p* = 0.91.

#### 2.2.4. Perspective-Taking Ability (PTA) Task

**Figure 3:**
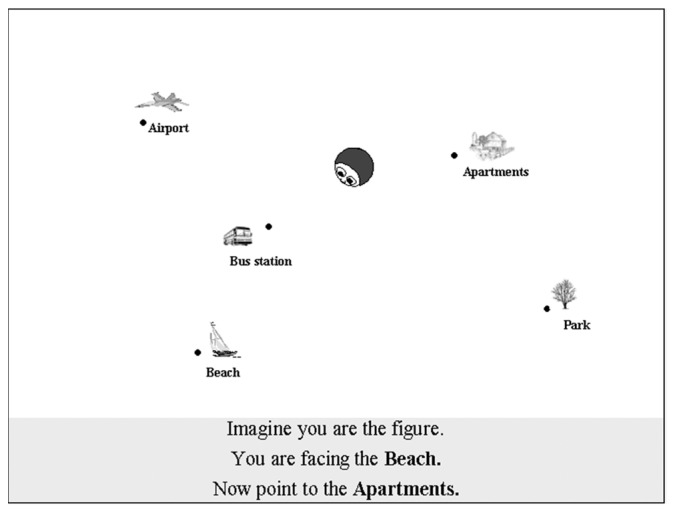
An example of a trial from the perspective-taking task.

The purpose of this task is to measure participants’ ability to imagine how the array of objects will look from a novel perspective. The task consists of 72 trials, during which scenes and objects were presented to the participant on a standard computer screen (Figure 3).

On each trial, a figure representing a character’s head indicated the starting location where participants were to imagine themselves standing. After a short delay, a flashing red dot in place of the black dot indicated which landmark was to be pointed to. Participants were to indicate the direction to a target location by clicking one of eight directional arrows shown on the same screen, which indicated directions in increments of 45° from 0° to 315°. Both accuracy and response time were recorded. Participants were instructed to imagine transforming their actual perspective (i.e., an aerial perspective of the character and the town) to that of the character’s perspective, and then the participants were to imagine pointing to the target from the character’s perspective. The imagined orientations in the perspective-taking version varied from 100° to 260° (relative to the upright direction) in increments of 20°. Angles less than 100° and more than 260° were not used for imagined headings because previous research [52] has shown that observers usually used strategies other than perspective-taking strategies for those angles (e.g., analytical strategies or tilting the head to ‘see’ the angle).

#### 2.2.5. Mental Rotation (MR) Task

**Figure 4:**
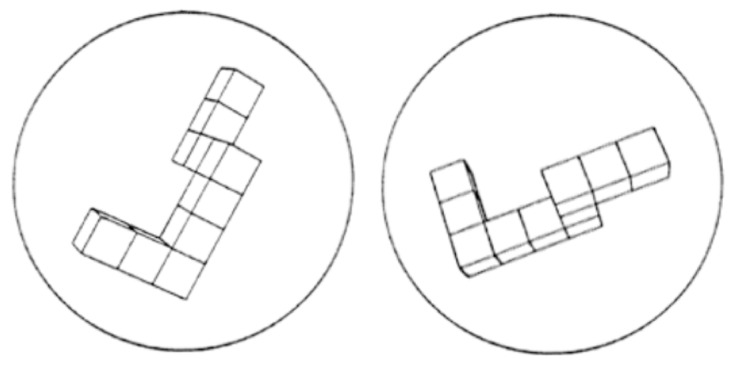
An example of a trial from the mental rotation task.

This task assessed mental rotation ability, requiring participants to mentally manipulate an object, employing object-to-object spatial representations from the fixed perspective of the viewer. The task administered was the MM Virtual Design Mental Rotation Test Version 1.1, a computerized version of the original task developed by Shepard and Metzler [53]. On each trial, participants were presented with two-dimensional pictures of a pair of three-dimensional geometric figures (Figure 4).

Participants were asked to determine if the pair of figures were the same or different and responded by clicking either the left or right mouse key. Participants completed 8 practice trials with feedback, followed by 36 experimental trials without feedback. Half of the trials presented different pairs of figures, while the other half presented pairs of the same figure, rotated at 7 possible angles between 40° and 180°, either in the picture plane or in depth.

#### 2.2.6. Procedure

The experiment consisted of three main parts: a triangle completion task, a route traversal, related tasks (R-PDT, drawing sketch maps), and lab-based computer tasks. All participants ended the study with lab-based computer tasks. Each participant was tested individually. For participants with even-numbered IDs, the experimenter began the study by leading them through the route at the faculty of engineering. At the route’s endpoint, the participants completed the sketch-map drawing task and then performed the R-PDT. Although requesting participants to draw their sketch-map before the R-PDT task might have led to better performance on R-PDT (as spatial information about the route layout becomes explicit after the drawing task), counterbalancing the order of the sketch-map drawing task and R-PDT was not possible. Administering R-PDT with a disorientation paradigm before sketch map tasks would have affected the quality of the sketch maps, especially for those participants who rely on path integration, making their classification impossible. After completing the final task, the participant and experimenter traveled to another location on campus (a 10 min walk away), where they undertook the triangle completion task, followed by informal interviews with the experimenter about their navigational strategies, environmental features they paid attention to while learning the route, and their memories of the route. The participants then walked to the experimental lab (a 5 min walk away), where they did the computer tasks. For participants with odd-numbered IDs, the order of the first two tasks was reversed (i.e., they did the triangle completion task followed by the route traversal, sketch map drawing, R-PDT, and the computer tasks). All participants performed the PTA task at the lab, followed by the MR task. Measures of accuracy and response time were recorded for the small-scale assessments, while mean absolute deviations for Time 1 and Time 2 were recorded for the R-PDT. Upon completion of the final task of the experiment, participants were reimbursed or given study credits. A debriefing statement was subsequently sent to their email. The entire experimental session lasted about 1.5 h.

### 2.3. Results

Three participants were removed from the statistical analyses as outliers, as their performance on the R-PDT (before disorientation) was near chance level (mean pointing accuracy deviating more than 160 degrees from the direction). Thus, data from a total of 68 remaining participants were analyzed in this study.

#### 2.3.1. Sketch-Map Categorization

**Figure 5:**
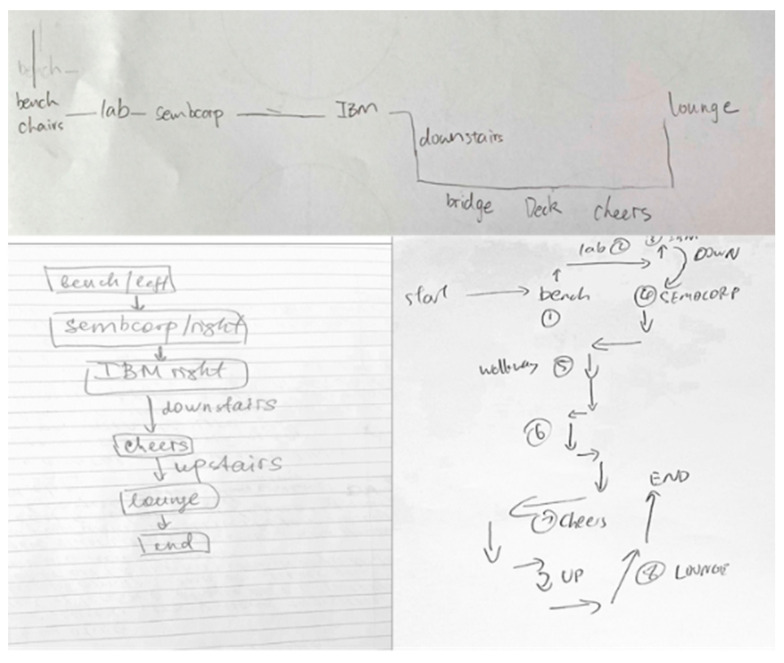
A representative sketch map from the procedural category.

**Figure 6:**
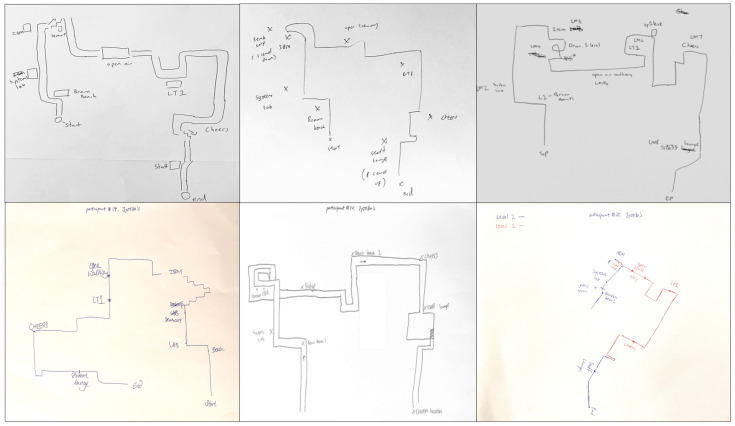
Representative sketch maps from the allocentric-survey category.

**Figure 7:**
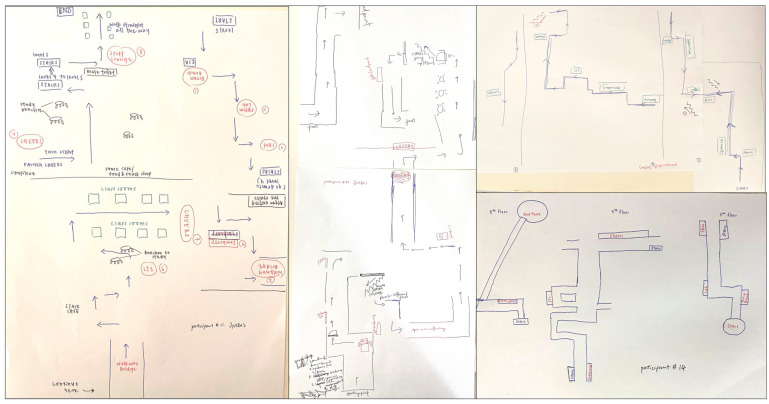
Representative sketch maps from the egocentric-survey category.

Four participants drew maps with few depictions of the route and landmarks, making them ineligible for further classification. These participants’ data were removed from further analyses. Two coders independently analyzed and categorized the remaining 64 sketch maps (26 males) into three categories: (i) procedural route maps; (ii) allocentric-survey maps; and (iii) egocentric-survey maps. In the categorization of the sketch maps, an agreement between the two coders was 94% at the outset, and any disagreement was discussed until a consensus was reached. The categorization method adopted in this study was similar to that of Blajenkova, Motes et al. [30], and Zhong and Kozhevnikov [33]. Specifically, the sketch maps that represented linear, non-spatial representations of navigational procedure (e.g., “here turn left”, “here go down one floor”) or the sequence in which the landmarks were encountered, without any attempts to capture the spatial relationships between the landmarks on the route, were categorized as *procedural maps* (*N* = 20; 18 females) (Figure 5). In contrast, sketch maps that displayed a coherent spatial structure of the route with spatial relationships between the landmarks and geometry of the route approximating those presented in the official floor plan were classified as survey maps. Furthermore, the sketch maps that represented the spatial layout of the route and its surrounding environment holistically (as a global unit rather than segmented representations) from a single allocentric perspective (usually top-down, although a few maps presented the route from a side view) were categorized as *allocentric-survey maps* (*N* = 22; 10 females) (Figure 6). The sketch maps representing the route and its surrounding environment from several different perspectives (as indicated by the separation of the route into three segments traversed at different levels, each of them portrayed from a first-person perspective aligned with the participant’s heading direction on that route segment) were categorized as *egocentric-survey maps* (*N* = 22; 8 females) (Figure 7).

**Figure 8:**
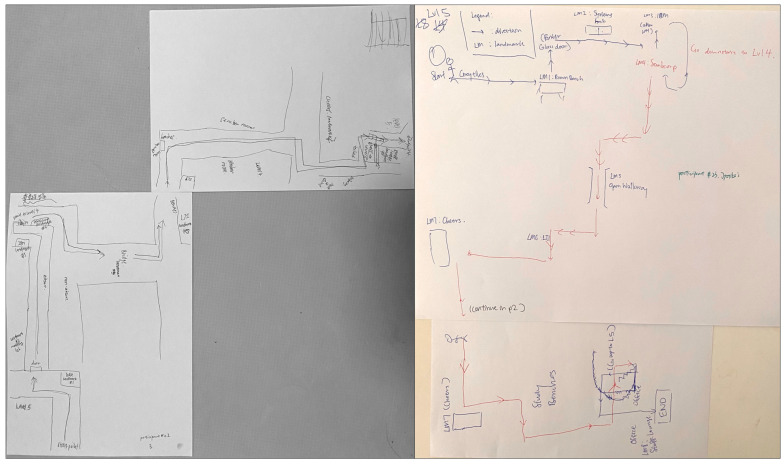
Egocentric sketch maps drawn on two pieces of paper, subsequently attached to each other.

This is in contrast to the majority of allocentric maps, which represent environmental features of landmarks and route segments resting on a single level. Similar to allocentric-survey sketch maps, they largely preserved the spatial relationships between the landmarks and the distances between them. Egocentric-survey sketch maps were larger than two other types of sketch maps, and six egocentric-survey mappers asked for an additional sheet of paper to complete their drawing (Figure 8).

After being categorized into three categories, the three types of sketch maps were examined further with respect to their common and distinct features. First, there was a significant difference in the frequencies of landmarks, including the 8 landmarks that were pointed out by the participants and the other landmarks they passed along the route, depicted (in either visual or verbal format) on the sketch maps of different types, *F*(2, 63) = 47.24, *p* < 0.001, post hoc comparisons using Tukey HSD revealed that egocentric-survey maps depicted more landmarks (M = 20.00, SD = 6.80) than allocentric-survey maps (M = 8.09; SD = 2.40), *p* < 0.001, and procedural/route maps (M = 10.01; SD = 1.65), *p* < 0.001, while there was no difference between the procedural and allocentric-survey maps, *p* = 0.29). Second, as seen in Figure 6, Figure 7 and Figure 8, there was a difference between the types of landmarks depicted on the sketch maps of different categories. Similar to the results reported in Blajenkova, Motes et al. [30], while procedural mappers depicted mostly turning point landmarks, egocentric-survey mappers depicted both turning and non-turning point landmarks on the route as well as those landmarks that were visible from but located off the route. Moreover, egocentric-survey maps were more likely to contain landmarks drawn from the orientation they were seen in during the route traversal, while the landmarks on allocentric-survey maps, in most cases, were labeled verbally or drawn as blocks without any particular orientation. The results suggest egocentric encoding of landmarks and their locations by the egocentric-survey mappers and orientation-free landmark encoding by the allocentric-survey mappers. Moreover, 12 out of 22 allocentric-survey maps were structured by double lines (e.g., the left sketch map on the top and the middle map on the bottom of Figure 6), suggesting that allocentric-survey mappers paid particular attention to the route geometry and environmental boundaries while encoding the route.

#### 2.3.2. Descriptive Statistics

The accuracy scores for each experimental task for all types of map sketchers are shown in Table 2. For the R-PDT task, the absolute error (in degrees) between the pointing directions indicated by the participants and the actual angle of the landmark’s bearing was calculated and then averaged for the four trials pre-disorientation (Time 1) and the four trials post-disorientation (Time 2). Similarly, the final scores for the triangle completion task were calculated by averaging absolute heading errors (in degrees) across the seven trials.

For MR and PTA tasks, to avoid confounds arising from speed-accuracy trade-offs, often reported during the performance of spatial tasks, we created a measure of processing efficiency by dividing each subject’s proportion of correct responses by their ln(RT). A logarithmic transformation was used to normalize the RT data that were positively skewed. Thus, the efficiency measures we report refer to the number of correct responses made by the participant in one unit of time (ln seconds).

Regarding accuracy and efficiency scores, one way ANOVAs (see Table 2) revealed significant differences between the three groups of map sketchers in the performance of R-PDT Time 1, PTA, and the triangle completion task. All further post hoc comparisons of R-PDT Time 1, triangle completion, MR, and PTA tasks were performed using the Tukey *HSD* test. For R-PDT time 1, there was a significant difference in pointing accuracy between procedural and egocentric-survey mappers (*p* = 0.03), a marginal difference between allocentric-survey and procedural mappers (*p* = 0.07), but no significant difference between allocentric and egocentric mappers (*p* = 0.93). For PTA efficiency, the only significant difference was between procedural and egocentric-survey mappers (*p* = 0.01); all others had *p* > 0.25. For the triangle completion task, egocentric-survey mappers were the most accurate, and their performance was significantly better than both procedural (*p* = 0.03) and allocentric-survey mappers (*p* = 0.001), while there was no difference between allocentric and procedural mappers (*p* = 0.55). For the MR task, procedural mappers performed significantly worse than egocentric-survey mappers (*p* = 0.03), while there was no difference between the two groups of survey mappers (*p* = 0.77).

Furthermore, a two tailed Pearson bivariate correlation analysis was conducted to evaluate the relationship between the participants’ R-PDT performance before disorientation and other measures (i.e., pointing accuracy on the triangle completion task, PTA task efficiency, and MR efficiency). For these analyses, we used R-PDT Time 1 and Time 2 accuracy scores, calculated by subtracting mean pointing errors during Time 1 and Time 2, respectively, from 180°, so that the higher scores reflected better performance. Similarly, the final scores for the triangle completion task were calculated by subtracting the mean heading error from 180°. The results are presented in Table 3. The R-PDT score before disorientation (Time 1) was correlated marginally with triangle completion task accuracy (*p* = 0.08) and significantly with PTA efficiency (*p* = 0.02) but not with MRT (*p* = 0.58). There was a significant correlation between MR and PTA tasks (*p* < 0.001).

Linear regression with R-PDT Time 1 as the dependent variable and pointing accuracy on the triangle completion task, PTA, and MR efficiency as predictors was only marginally significant, *F*(3, 63) = 2.54, *p* = 0.06, R-square = 0.12, with PTA efficiency being the only significant predictor of R-PDT Time 1 performance (*p* = 0.047).

#### 2.3.3. Effects of the Disorientation Paradigm

**Figure 9:**
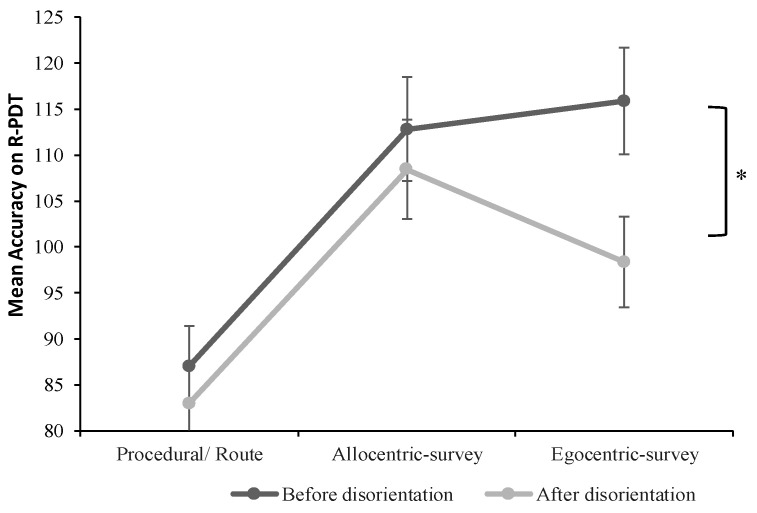
The mean pointing accuracy on the R-PDT task before and after disorientation by Map Type. Error bars show ± 1 SEM, * *p* < 0.05.

To compare the pointing error between the different navigator types on the R-PDT trials pre- and post-disorientation, a 2 × 3 mixed ANOVA was performed with Time (Time 1, Time 2) as the within-subject variable and Map Type (Procedural, Allocentric-survey, and Egocentric-survey) as the between-subject variable (Figure 9).

The main effect of Time was only marginally significant, *F*(1, 61) = 3.28, *p* = 0.07, so participants’ performance on the R-PDT tended to worsen after disorientation. The main effect of Map Type was not statistically significant; *F*(2, 61) = 1.99, *p* = 0.15. However, the interaction between Time and Map Type was significant; *F*(2, 61) = 3.65, *p* = 0.03. As indicated previously, procedural/ route sketchers performed significantly worse than allocentric-survey and egocentric-survey sketchers before disorientation. There was no difference between all three types of map sketchers after disorientation. A simple effect test of Time at each level of Map Type was conducted, which was of main interest as the disorientation was predicted to affect only the pointing accuracy of egocentric-survey mappers. Out of the three groups, only participants who formed egocentric-survey representations were significantly affected by the disorientation, showing lower pointing accuracy at Time 2 than at Time 1, *t*(21) = 3.95, *p <* 0.001. Allocentric-survey sketchers did not show a difference between R-PDT Time 1 and R-PDT Time 2, *t*(21) = 0.9, *p* = 0.49. Similarly, procedural/sketchers did not demonstrate significantly different pointing accuracy between Time 1 and Time 2, *t*(19) = 0.66, *p* = 0.41. Thus, egocentric-survey mappers seemed more susceptible to having their directional knowledge disrupted by disorientation than allocentric and procedural sketchers.

#### 2.3.4. Survey Responses

After completing R-PFT tasks, all participants were interviewed regarding their navigational strategies (see Table 4). While the reports from the procedural mappers explicitly mentioned attending to and remembering landmarks with the turns associated with them, the reports from both the allocentric-survey mappers and egocentric-survey mappers reflected strong considerations for the mapping of spatial relations, either between landmark locations (allocentric-survey mappers) or between the moving body and surrounding landmarks (egocentric-survey mappers). Both groups of survey mappers also reported attending to visual information while learning the route. However, while allocentric-survey mappers reported attending to route boundaries and landmark locations in relation to each other and the route, egocentric-survey mappers reported paying attention to orientation-specific visual appearances of the landmarks. Furthermore, as can be seen in Table 4, allocentric-survey mappers described piecing together route segments and mapping spatial relations between the landmarks encoded in an orientation-free manner while forming a mental representation of the route; in contrast, egocentric-survey mappers reported forming their representations of the route by tracking their position and orientation with references to salient landmarks encoded in an orientation-specific manner. 

### 2.4. Discussion

The results of Experiment 1 suggest that in addition to procedural, route-based representations, there are two distinct types of survey-based representations: allocentric-survey and egocentric-survey representations. In contrast to both groups of survey mappers, the procedural group depicted on their sketch maps mainly had the turning point landmarks organized in a linear sequence (i.e., preserving temporal but not spatial relations between the landmarks). Although both types of survey mappers presented relatively accurate spatial representations of the route on their sketch maps, in addition to representing the spatial layout from a third-person perspective by the allocentric mappers and from a mixture of egocentric perspectives by the egocentric mappers, these two types of survey mappers included in their sketch maps different amounts and types of visual information. Consistent with their verbal reports and the use of “egocentric language”, the egocentric-survey mappers depicted significantly more landmarks than the allocentric-survey mappers and of larger size, in most cases drawn from the orientation they were seen on the route. In contrast, the allocentric-survey mappers paid particular attention to the route geometry and environmental boundaries and portrayed relatively fewer landmarks than the egocentric-survey mappers, usually presented in an orientation-free manner (either verbally or as orientation-free blocks). Consistent with our hypothesis that the two types of survey maps are different in the use of frames of reference (multiple frames of reference vs. a unifying allocentric one), in their sketch maps, only the egocentric-survey mappers sketched the route segments on the different floors separately, while the sketch maps of the allocentric-survey mappers were holistic, drawn on a single level.

Overall, the verbal reports and sketch maps of these two types of survey mappers suggest that the allocentric-survey mappers mainly paid attention to the route geometry and spatial relations between landmarks (i.e., preserving spatial relations), while the egocentric-survey mappers relied on both the landmark temporal sequence and their spatial relations to track their position on the route (i.e., preserving both temporal and spatial relations).

Furthermore, while both egocentric and allocentric mappers were significantly more accurate than the procedural group on the R-PDT task, we found no difference between egocentric- and allocentric-survey mappers on this task before the disorientation. Although these results might seem to weaken our conclusions, as previous research has shown that the employment of egocentric representations facilitates locating and pointing to landmarks quickly and accurately [54], it should be considered that our research design required administering the R-PDT task after asking participants to draw their sketch maps of the environment. As spatial information about the route layout becomes explicit after sketching the route, this could have led allocentric-survey mappers to improve their performance on the R-PDT task, resulting in no significant differences in accuracy between the two groups of survey mappers. Importantly, while neither procedural nor allocentric-survey mappers were affected by disorientation, egocentric-survey mappers exhibited a significant decrease in accuracy on the R-PDT task after the disorientation. These results suggest that only the egocentric-survey group relied on the path integration strategy, as idiothetic cues from vestibular and proprioceptive systems are error-prone and the most susceptible to external interference, such as disorientation [42,55]. Previous physiological studies used the disorientation procedure in an attempt to enhance the control of visual cues over idiothetic signals while the rat was searching for a reward location (see [56] for a review). These attempts, however, turned out to be largely unsuccessful during the exploration of novel environments, as visual cues might exert control over the path integration only after the animal has learned a stable mapping between the visual information and the head direction information [42,56]. As participants in our experiment did not have enough experience with the route to form a strong association between the allothetic and idiothetic cues in the environment, it would explain why our egocentric-survey mappers showed impaired performance on the R-PDT task after disorientation as compared to the allocentric-survey mappers, who mainly rely on allothetic signals (i.e., multiple landmark locations) while learning the route. Additional evidence that egocentric-survey mappers rely more on path integration than the other two groups comes from the results demonstrating that egocentric-survey mappers exhibited significantly more accurate performance on the triangle completion task, measuring one’s ability to path integrate while relying entirely on idiothetic cues.

While most previous studies have largely ignored individual differences in path integration, the results of Experiment 1 demonstrate that they are critical to navigation performance as they support the formation of egocentric survey-based environmental representations. At the same time, we found that both groups of survey mappers performed significantly better on the computerized PTA and MR tasks than procedural mappers, suggesting that “small-scale” spatial ability is also an essential factor in forming survey-type spatial knowledge. Consistent with previous research [57], the PTA task appeared to be the most reliable predictor of navigation behavior, as it accounted for more variance when included in regression along with MRT and the triangulation task. In contrast to the triangulation task, however, the PTA has been unable to discriminate between allocentric- and egocentric-survey mappers, possibly because accurate assessments of path integration capacities require more realistic viewing environments (e.g., immersive virtual reality), rich with both allothetic and idiothetic information.

## 3. Experiment 2

### 3.1. Methods

#### 3.1.1. Participants

Forty-eight participants (12 males) aged 19 to 36 (M age = 22, 25 y.o., SD = 2.91) were recruited through the Psychology department’s research participation program at the National University of Singapore in exchange for course credit and/or monetary reimbursement. Similar to Experiment 1, an a priori power analysis with an effect size set to 0.30, an alpha level of 0.05, a correlation between repeated measures of 0.5, and statistical power of 80% was conducted, yielding a minimum sample size of 48 participants. To ensure a balanced number of participants in each group of navigators (procedural, allocentric survey-based, and egocentric survey-based), the self-reported Navigational Strategy Questionnaire (NSQ, Zhong and Kozhevnikov [33]) assessing individual differences in different navigational strategies was administered to the participants prior to the experiment. All participants were recruited based on the prerequisite of being unfamiliar with the premises around the NUS School of Engineering, where the experiment was taking place.

#### 3.1.2. Route Traversal

All the participants were led individually by the experimenter on the same route as in Experiment 1 (Figure 1). The procedure and instructions given to the participants on the route were identical to those described in Experiment 1.

#### 3.1.3. Tasks and Materials

The sketch map, PTA, and MR tasks administered to the participants in this experiment were identical to those described in Experiment 1.

##### Imagined-Pointing Direction Task (I-PDT)

The I-PDT was adapted from a judgment of relative directions task (J-RDT) that requires judgments of directions relative to specific imagined orientations or viewpoints in large-scale space [58,59]. In this task, the participants were asked to imagine standing at a particular landmark, facing another landmark, and pointing to a third target landmark based on the imagined orientation. All the landmarks used during this task were those of the 8 landmarks pointed out to the participants during the route traversal.

On each trial, the names of three landmarks were presented on a computer screen. The participants were instructed to imagine themselves standing at the location of the first landmark specified by the caption “STAND AT” at the top of the screen, mentally reorienting themselves to face a second landmark specified by the caption “FACING” in the middle, and then point to a third landmark specified by the caption “POINT TO” at the bottom.

The names of 8 landmarks pointed out en route were applied in different combinations of threes. IPDT trials were designed in such a way that the imagined heading (i.e., the angular difference between the starting leg of the route and the target heading, which is the vector connecting the landmark indicated by “STAND AT” and the landmark indicated by “POINT TO”) changed systematically from 100° to 180° with an absolute interval of 20° (i.e., 100°, 120°, 140°, 160°, and 180°). Four keys (i.e., 1, 3, 7, and 9), positioned on the right panel of the computer keyboard, were available for the participants to indicate their pointing directions [i.e., Back-Left (BL), Back-Right (BR), Front-Left (FL), and Front-Right (FR)], respectively.

There were 24 experimental trials of I-PDT for each participant; 12 of them were performed with a distractor task (spatial working memory, SWM) and 12 without the distractor task, in a randomized order. As both imagined heading and pointing directions contribute to the task’s difficulty [52], we matched the trials with the SWM distractor task as closely as possible to those without the SWM distractor task by the imagined heading and pointing directions shown in Table 5. The participants’ accuracy and response time in each trial were recorded.

##### Spatial Working Memory (SWM) Distractor Task

A distractor SWM task was based on the paradigm used by Yokoyama, Kato, et al. [60]. This task involved red circles flashing at two different points on the screen for 500 ms, one after another.

**Figure 10:**
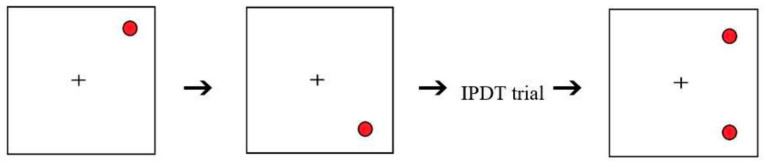
A sample dual-task paradigm trial sequence is shown in this figure. The ‘+’ is the fixation cross that is briefly flashed on the screen before the participants are shown two red circles flashing at two different points on the screen for 500ms, one after the other. This is followed by the I-PDT experimental trial. Finally, in the case shown above, the participant is shown a screen that has red circles arranged in a configuration that is the same as the combination of the initial stimuli. The alternative case (not pictured above) is when the configuration of the circles is different from the combination of the initial stimuli shown.

This was followed by one I-PDT experimental trial, as shown in Figure 10.

After participants responded on the I-PDT trial, two flashing circles appeared on the screen in a configuration that was either the same or different from the combined configuration of the initial flashing circles. Participants were required to respond by pressing either the “S” or “D” keys to indicate “same” or “different”, respectively. To ensure above-chance level performance on this task, the distractor task trials were kept simple, with the circles in the stimuli and the resulting configuration appearing only at the four corners of the screen. 

##### Procedure

The experiment consisted of two parts: (1) route traversal followed by the sketch map task, and (2) I-PDT, PTA, and MR tasks completed in the lab. Each participant was run individually. Participants and the experimenter met at a location close to the starting point of the route, where they were briefed on study instructions, signed the consent form, and then led to the starting point of the route. At the endpoint of the route, the participants proceeded to complete the sketch-map drawing task, after which the participants and experimenter traveled to another location on campus (a 10 min walk away), where they did the I-PDT (12 trials with the SWM distractor task and 12 trials without the distractor in a randomized sequence), PTA, and MR tasks. After completing the tasks, the participants were compensated via course credits or monetary rewards. They were subsequently debriefed. On average, each participant took 1 h and 30 min to complete the experiment.

### 3.2. Results

One participant was removed from the data analyses due to below-chance performance on a secondary SWM task (<0.5), and two other participants had unusually long RTs (average RT > 25 s) on I-PDT and PTA tasks, respectively. Next, based on the same categorization criteria described in Experiment 1, two coders independently analyzed and categorized the remaining sketch maps into three categories, according to the same criteria as described in Experiment 1. Two participants drew maps ineligible for further classification. The resulting 45 sketch maps were categorized into the following categories: (i) 15 procedural route maps (twelve females), (ii) 12 allocentric-survey maps (seven females), and (iii) 18 egocentric-survey maps (ten females). In the categorization of the sketch maps, agreement between the two coders was 92% at the outset, and any disagreement was discussed until a consensus was reached.

#### 3.2.1. Descriptive Statistics

The accuracy scores for each experimental task for all types of map sketchers are shown in Table 6. For the I-PDT task (with and without SWM), the number of correct responses [i.e., pointing to the correct quadrant (FL, FR, BL, or BR) where the target was located] was calculated and averaged for the 12 trials without an SWM task (I-PDT 1) and the 12 trials with a concurrent SWM task (I-PDT 2). 

With regards to accuracy and efficiency scores, one way ANOVAs (see Table 4) revealed significant differences between the three groups of map sketchers in the performance of I-PDT-1 and I-PDT2 accuracy, I-PDT2 RT, PTA, and MR tasks. All further post hoc comparisons were performed using the Tukey *HSD* test. For both I-PDT with and without the SWM distractor, there was a significant difference in pointing accuracy between procedural and egocentric sketchers (*p* < 0.005), as well as between procedural and allocentric sketchers (*p* < 0.05), but no significant difference between allocentric- and egocentric-map sketchers (*p* > 0.60). In terms of I-PDT RT w/o the distractor, there was no difference between all three groups of navigators (*p* > 0.21). For PTA efficiency, the only significant difference was between procedural and egocentric sketchers (*p* = 0.04); all others had *p* > 0.78. For the MR task, the procedural mappers performed significantly worse than allocentric-survey mappers (*p* = 0.002) and marginally worse than egocentric-survey mappers (*p* = 0.08), while there was no difference between the two groups of survey mappers (*p* = 0.17). Furthermore, a linear regression with I-PDT efficiency w/o SWM distractor [calculated as I-PDT proportion correct divided by ln(RT)] as the dependent variable and PTA and MR efficiency as predictors was significant, *F*(2, 44) = 2.54, *p* < 0.001, R-square = 0.30, with both PTA (*p* = 0.004) and MR (*p* = 0.003) efficiency being significant predictors of I-IPDT w/o SWM, explaining together 30% of the variance.

#### 3.2.2. Effects of Concurrent SWM Tasks

To compare the pointing accuracy on I-PDT with and without a concurrent SWM task between different navigator types, a 2 × 3 mixed ANOVA was performed with Condition (with SWM vs. w/o SWM) as a within-subject variable and Map Type (Procedural, Allocentric-survey, and Egocentric-survey) as a between-subject variable. Overall, the main effect of Condition was not significant, *F*(1, 42 < 1, *p* = 0.37. The main effect of Map Type variable was significant, *F*(2, 42) = 7.37, *p* = 0.002; pairwise comparisons (Tukey HSD) revealed significantly less accurate performance of procedural sketch mappers than either allocentric-survey mappers (*p* = 0.02) or egocentric-survey mappers (*p* = 0.002), while there was no difference between the allocentric-survey and egocentric-survey mappers (*p* = 0.81). The interaction between Condition and Map Type was not significant, *F* < 1, *p* = 0.64.

**Figure 11:**
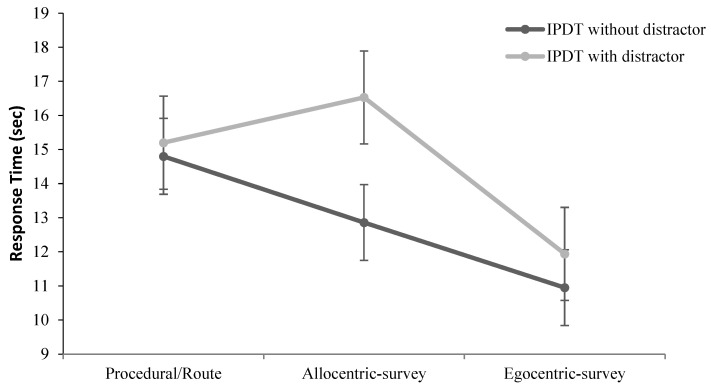
The mean RT of IPDT without and with a distractor, arranged by navigator group. Error bars show ± 1 SEM.

Next, to compare I-PDT RT with and without a concurrent SWM task between different navigator types, a 2 × 3 mixed ANOVA was performed 2 × 3 with Condition (with SWM, w/o SWM) as the within-subject variable and Map Type (Procedural, Allocentric-survey, and Egocentric-survey) as the between-subject variable. The results are presented in Figure 11.

The main effect of the Condition was significant, *F*(1, 40) = 9.96, *p* = 0.004; participants exhibited significantly faster RT without a SWM distractor. The main effect of the Map Type variable was significant *F*(1, 40) = 4.00, *p* = 0.03; pairwise comparisons (Turkey) revealed significantly faster RT in the group of egocentric-survey mappers than in procedural mappers (*p* = 0.01) or allocentric-survey mappers (*p* = 0.03), while there was no difference in RT between the allocentric-survey mappers and egocentric-survey mappers (*p* = 0.83). Lastly, the interaction between Condition and Map Type was also significant, *F*(2, 41) = 3.25, *p* = 0.049. A simple effect test of Map Type at each level of Condition was further conducted. At the I-PDT w/o SWM task, significantly faster RT was found in the group of egocentric-survey mappers as compared to the group of procedural mappers (*p* = 0.016), while there was no difference in RT between the allocentric-survey mappers and egocentric-survey mappers (*p* = 0.20). At the I-PDT with the concurrent SWM task, egocentric mappers exhibited faster RT as compared to procedural mappers (*p* = 0.007) or allocentric mappers (*p* = 0.036). Furthermore, a simple effect test of Condition at each level of Map Type was also conducted, which was the interest of this study as the presence of SWM distractors was predicted to influence more allocentric-survey mappers than egocentric-survey mappers. Out of the three navigator groups, the significant difference in RT between I-PDT with SWM and IPDT without SWM was only for allocentric-survey mappers *t*(11) = 3.44, *p* = 0.005, suggesting that they were most affected by the concurrent SWM distractor. The differences between I-PDT with SWM and I-PDT w/o SWM for either procedural or egocentric-survey mappers were insignificant, *t*(14) = 0.39, *p* = 0.70, and *t*(15) = 1.42, *p* = 0.17, respectively.

### 3.3. Discussion

As required by the I-PDT task, judgments of relative directions are commonly used to assess allocentric spatial relations related to spatial memory and are facilitated when one uses an enduring abstract spatial representation [45]. Thus, we expected allocentric-survey mappers to perform better on this task when no concurrent distractor was present, as compared to egocentric-survey mappers. However, consistent with the results of Zhong and Kozhevnikov [33], in terms of accuracy, both allocentric- and egocentric-survey mappers were equally successful at retrieving information on spatial relations from their survey knowledge to solve the I-PDT task without the concurrent SWM distractor. Similar to Experiment 1, where the R-PDT task was administered to the participants after sketching the route maps, in Experiment 2, the I-PDT task was also performed after completion of the map drawing task, which could have helped egocentric-survey mappers perform more accurately on this task by making their knowledge about the spatial relations between the landmarks more explicit. Similarly, we did not find allocentric-survey mappers to respond significantly faster than egocentric-survey mappers without a distractor. This is in contrast to Zhong and Kozhevnikov’s [33] study, which used a similar paradigm and found egocentric-survey mappers to respond significantly faster than allocentric-survey mappers on the J-RPD task. Zhong and Kozhevnikov [33] suggested that since the egocentric-survey group encoded multiple egocentric views of landmarks aligned with the reference direction (i.e., an egocentric direction aligned with a line of objects/landmarks experienced during route traversal), the stored egocentric spatial relations were directly retrieved during the I-PDT trials, thus facilitating their overall speed of responses. However, many of the I-PDT trials in our study required the participant to imagine a new perspective (an imagined heading) that was not aligned with the reference direction (i.e., an experienced view during the route traversal). Therefore, mere retrieval of stored egocentric relations was insufficient to perform successfully on these trials, as they required additional mental transformation (i.e., spatial reorientation to a new imagined heading) from both allocentric- and egocentric-sketch mappers.

Importantly, Experiment 2 demonstrates that while there was no difference in the I-PDT task between allocentric- and egocentric-survey mappers without the SWM distractor, the performance of allocentric-survey mappers became significantly slower when a concurrent SWM task was employed. At the same time, although the overall response time of the procedural mappers was significantly longer than that of the egocentric- and allocentric-survey mappers, neither the procedural group nor the egocentric-survey mappers were slowed down by the distractor task. These findings suggest that only allocentric-survey mappers rely on spatial working memory while retrieving spatial relations. Indeed, empirical evidence suggests that allocentric spatial memory is conditioned by visual-spatial working memory capacity [61,62,63]. For example, De Beni, Pazzaglia, et al. [62] demonstrated that recall of spatial text was more readily disrupted and slower response times were noted by performance on a concurrent spatial task (i.e., tapping) than via articulatory suppression. Similarly, Garden, Cornoldi, et al. [63] reported that on the route-learning task, the survey knowledge group displayed more errors and longer pauses when engaged in spatial tapping than articulatory suppression, whereas the reverse was true for the low survey (i.e., procedural) knowledge group. Although the above studies did not explicitly distinguish between allocentric- and egocentric survey-based mappers, their findings suggest that spatial working memory is the primary cognitive resource to encode and retrieve the spatial locations of objects in the environment.

Finally, we observed considerable individual differences in performance on the I-PDT task. While there was no difference in the performance of allocentric- and egocentric-survey mappers on the I-PDT task without distractor, MR, or PTA, procedural mappers performed significantly worse. Furthermore, our results showed that performance on the I-PDT task without the SWM distractor was significantly predicted by both PTA and MR efficiency. One possible explanation is that there are two strategies to solve the I-PDT items successfully: either by performing an egocentric (perspective-taking) spatial transformation (imagining reorientation to a new imagined heading) or by performing an allocentric (mental rotation) transformation (mentally rotating an allocentric cognitive map of an environment until the heading to be imagined would match the facing direction).

## 4. General Discussion

The main goal of this research was to investigate whether egocentric-survey representations form a qualitatively different type of survey-based knowledge and whether path integration strategies contribute to their formation. The results of Experiments 1 and 2 demonstrate double dissociation between the navigational strategies underlying the formation of allocentric- and egocentric survey-based representations via the disorientation and dual-task paradigms. Specifically, we provided evidence that individuals who generated egocentric-survey representations were the only group affected by disorientation but, not a secondary spatial working memory task. At the same time, vice versa was true for the allocentric-survey mappers. Altogether, the findings provide evidence that egocentric-survey mappers relied primarily on path integration, resulting in the generation of orientation-specific environmental representations that preserved an accurate spatial layout of the route. In contrast, allocentric-survey mappers mainly relied on allocentric/map-based navigation, which involves the integration of the route features into an allocentric-survey representation in spatial working memory. Their allocentric survey-based representations captured the spatial layout of the route from a global perspective. 

Due to the rapid accumulation of spatial errors during path integration, many previous studies have considered path integration to be the least useful navigational strategy [64,65,66], which, except for cases where an environment is unfamiliar or lacks distinct environmental features, is usually used as a complement to piloting (see [67,68] for reviews). Indeed, research on human navigation has reported inaccurate encoding of path features (leg length and turning angles [69,70] and/or execution error (the error in the response, such as turning and walking a homebound trajectory [41,71]) during path integration. The majority of these studies, however, have applied the notion of path integration to navigation in either controlled, small-scale laboratory settings [13,69], where participants were deprived of visual and auditory cues about their position and orientation, or in virtual environments (e.g., [41,71]), where the optimal integration of vision and proprioception is still problematic. Such settings do not provide the participants with the same sources of information available during large-scale real-world navigation. Indeed, a significant improvement in encoding one’s body location was demonstrated in a real-world condition compared to immersive virtual reality [71], suggesting that the richness of allothetic cues and their integration with idiothetic signals might lead to more successful path integration. 

Importantly, the results of the current study suggest that the path integration strategy, combined with the egocentric encoding of landmarks and scenes, as it happens in the real world, is distinct from allocentric navigation. Although both egocentric- and allocentric-survey mappers in our study relied on allothetic information, they differed in the type of this information and how it was employed. Allocentric-survey mappers relied primarily on visual information about landmark locations and environmental boundaries. This type of information was suggested to play a major role during allocentric navigation in supporting the selection of a global frame of reference in relation to which the spatial relations between environmental features are encoded and organized in memory [72,73]. As for the egocentric mappers in our study, the multiple frames of reference they drew in their sketch maps were not dependent on visual information but rather “anchored” into the segments of the route they traversed, with each of the frames being aligned with the direction of motion at the corresponding route segment. This view on path integration is consistent with earlier computational models [6,74], according to which the hippocampal place cells and the head-direction cells appear to be part of a preconfigured network of quasi-independent spatial reference frame representations enabling internal representation of abstract spatial relationships. Viewpoint-specific visual information (e.g., landmarks and scenes) is then secondarily bound, by an associative synaptic modification, to location representations within each frame. Thus, according to these models, the role of visual information is only secondary, as it is not essential for maintaining an internal description of location and orientation but rather for initializing the location within the appropriate frame on subsequent visits and correcting for spatial error accumulating due to path integration within the frame [42,75]. Other experimental studies [73,76] further supported the view that visual information plays a primary role during piloting but not during path integration. For example, Mou and Wang [73] demonstrated that piloting, which primarily relies on the representations of the spatial relations between visible items (e.g., landmarks, features of a boundary) and the invisible target (which participants were asked to point to after physically walking a circuitous path without vision), was less efficient across boundaries (i.e., when the invisible target was located in a different room from where the objects’ locations were learned) than within boundaries. In contrast, navigation that relied only on path integration was not sensitive to boundary crossing.

Altogether, our findings cast doubt on the conclusions from previous studies that, while learning a route or exploring a novel environment without navigational aids, individuals form only one specific type of survey-based representation, either orientation-specific and transient egocentric [31,37,38,39] or orientation-free and enduring allocentric [15,45]. It is also unlikely that individuals form and later maintain both egocentric and allocentric representations of an environment, with the former being more precise and the latter coarser but enduring [55], or that they can flexibly switch between these representations according to the task requirement [55,77]. Our results suggest that these two types of survey representations are qualitatively different, and their formation primarily depends on individual differences in visual-spatial abilities and path integration capacities. As the egocentric-survey mappers performed significantly better than procedural and allocentric-survey mappers on the path completion task, our findings suggest that forming egocentric survey-based representations, in addition to relatively high visual-spatial abilities, requires the ability to rely on self-motion cues during navigation. It is unlikely that when presented with environmental challenges (e.g., removal of salient landmarks, low visibility, or disorientation) or repeated exposure to the same environment, an individual will switch from one survey-based representation to another. It might be possible, however, to maintain egocentric and allocentric representations at the same time when the route information was learned from multiple sources, such as not only from the direct exploration of the route but also from a map, as these different types of route learning might facilitate different encoding processes [78]. Further research into the relationship between egocentric and allocentric survey-based representations is needed to enhance our understanding of their functions and how these survey-based representations might interact and transform.

Furthermore, egocentric and allocentric survey-based representations cannot be considered superior or inferior to each other, or one being less coarse and more accurate than the other, as they represent qualitatively different types of survey-based representations, each with their advantages and drawbacks. Both support flexible navigational behavior, such as planning new routes and finding shortcuts. Egocentric survey-based representations cannot be viewed as coarser than allocentric ones, as although they do not afford a coherent and global view of the environment as allocentric representations, they encode not only spatial (locations) but also temporal (sequence) information about the landmarks and scenes encountered on the route, in addition to more detailed information about landmarks’ orientation-specific appearance. In addition, egocentric survey-based representations cannot be viewed as less enduring than allocentric ones, as although they are more prone to disorientation compared to allocentric ones, they are less sensitive to any interference that might demand additional spatial working memory resources, and they might be affected less by boundary crossing compared to allocentric representations. In this respect, a recent Weisberg and Newcombe [79] study is of particular interest. Using a virtual-reality paradigm in which two routes were learned, the researchers identified two types of survey mappers: non-integrators who were better during within-route judgments and integrators who performed well on both within- and between-route judgments. According to the results of the current study, these groups seem to map on allocentric survey-based and egocentric survey-based mappers, respectively.

Overall, our findings suggest that the individual differences approach is critical for building functional and neurological models of spatial navigation in humans, as they vary considerably not only in how accurately they can form survey-based representations but also in the type of survey-based representations they form and the navigational strategies they rely on. In particular, future research should focus more on individual differences in path integration, which have received much less attention in spatial cognition literature than allocentric or procedural navigation [but see [80], who showed correlations of path integration abilities with gray matter volume in the retrosplenial cortex, hippocampus, and medial prefrontal cortex, and Zhong and Kozhevnikov [33], who designed a new self-report scale to access path integration ability]. 

From a neuroscience perspective, one approach to understanding how different navigational strategies and cognitive processes contribute to egocentric vs. allocentric survey-based representations is to look at the brain dynamics of different types of navigators using portable EEG devices. Previous research [81] reported stronger alpha-blocking in or near the right primary visual cortex of participants whose homing responses were compatible with the use of an egocentric reference frame. In contrast, participants who relied on the allocentric frame of reference exhibited stronger alpha-blocking of occipitotemporal, bilateral inferior parietal, and retrosplenial cortical areas. 

Another promising approach that might help shed light on how different types of survey-based representation are generated in the brain is to look at the contribution of the two input streams, the medial entorhinal cortex (MEC) and lateral entorhinal cortex (LEC), to the hippocampus, which support spatial and relational processing, respectively [82,83,84]. The MEC stream is connected to several other regions containing cell types carrying space- or motion-related signals, including grid cells, head direction cells, boundary cells, and others [85,86], and it is primarily dedicated to spatial processing, including path integration computation [6,23,87]. In contrast, LEC encodes information about items in the external world [82] and appears to support declarative memory and relational processing (the representation of concepts, objects, and events based on their common elements). Although LEC cells are not specialized for spatial processing, there is evidence that they are tuned for the egocentric bearing of the goal location in a goal-oriented task [88,89]. While the MEC stream sends the input to the hippocampus to generate a global allocentric spatial map of an environment, the LEC sends information about the landmarks in the environment and the sequence they encountered on the route [87], which might support allocentric and procedural navigation, respectively. At the same time, the integration of information from the MEC and LEC inputs by the hippocampus might support the generation of egocentric survey-type environmental representations. In the latter case, the MEC input, provided to the hippocampus by path integration mechanisms, may support configuring an accurate spatial layout. At the same time, the LEC may send to the hippocampus information about the appearances of landmarks and scenes on the routes, aligned with an egocentric direction (e.g., the direction of the navigator’s movement) at each segment of the route. Consistent with this idea, Maguire and Mullally [90] proposed that episodes are encoded as events that could still be spatially arranged. Indeed, the input from the MEC stream supporting path integration combined with the input from the LEC supporting the egocentric views of the scenes would allow moment-to-moment updating of a navigator’s position based on idiothetic information integrated with egocentrically encoded visual information at each segment of the route. The comprehensive understanding of the functions of the MEC and LEC streams and how information from both streams is integrated into the hippocampus, however, remains unclear. In addition, there is still an ongoing debate regarding the brain areas responsible for path integration, with several researchers proposing that it is performed in the parietal cortex, which provides inputs to the hippocampal system [91,92,93]. More neuroscience research is needed to identify brain networks underlying different navigational strategies, the formation of egocentric and allocentric environmental representations, their interaction, and their relation to the MEC and LEC streams. 

From a theoretical perspective, this study is important as it is the first to show that path integration, in conjunction with egocentric landmark processing, underpins the formation of a unique type of environmental representation—the egocentric survey-based representation. From a practical perspective, an understanding of individual differences in navigational strategies and environmental representations is beneficial to the design and application of in-vehicle navigation systems and air traffic control displays, as well as to personnel selection and training for jobs requiring high competence in either egocentric (e.g., air piloting, telerobotics, and robotic surgery) or allocentric (air traffic controllers) spatial knowledge. Knowing the benefits and vulnerabilities of each type of navigational strategy and corresponding environmental representations will help safeguard personnel from workplace failure. 

## 5. Conclusions

While most previous studies suggested that cognitive maps are allocentric and encode route segments holistically in a global coordinate system, the current study presents evidence that there is another type of cognitive map, which is egocentric and represents an environment from multiple frames of reference. The egocentric cognitive maps are “anchored” into the segments of the route, with each of the frames being aligned with the direction of the traveler’s motion at the corresponding route segment. This is in contrast to allocentric cognitive maps, which encode primarily visual information about landmark locations and environmental boundaries. The results demonstrate a double dissociation between the navigational strategies underlying the formation of allocentric- and egocentric survey-based representations. Specifically, only the individuals who generated egocentric survey-based representations of the route were affected by disorientation, suggesting they relied primarily on a path integration strategy combined with egocentric landmark/scene processing at each route segment. In contrast, only allocentric-survey mappers were affected by the secondary spatial working memory task, suggesting their use of allocentric navigation. Furthermore, our results suggest that although both types of cognitive maps represent an environment accurately, they are qualitatively different, and their formation primarily depends on individual differences in visual-spatial abilities and path integration capacities. 

## Figures and Tables

**Table 1:** Imagined heading and mean scores for each of the landmarks.

Time 1	Deviation from the Correct Angle		Time 2	Deviation from the Correct PD	
	Mean	SD		Mean	SD
LDMK #2	18°	23°	LDMK #5	30°	29°
LDMK #4	108°	48°	LDMK #3	105°	55°
LDMK #6	81°	36°	LDMK #1	80°	32°
LDMK #8	78°	30°	LDMK #7	74°	35°

**Table 2:** Descriptive statistics of the R-PDT mean error before disorientation (R-PDT Time 1) and after disorientation (R-PDT Time 2), the mean pointing error on the triangle completion task, the efficiency of MR and PTA, and ANOVA results.

Task	Procedural/Route Map Sketchers		Allocentric-Survey Map Sketchers		Egocentric-Survey Map Sketchers		ANOVA Results
	Mean	SD	Mean	SD	Mean	SD	*F*(2, 63)
(i) R-PDT Time 1	87.09	27.93	67.17	27.89	64.14	29.26	3.98 *
(ii) R-PDT Time 2	82.98	25.45	71.56.44	34.84	81.66	30.50	0.89
(iii) MR efficiency	0.42	0.09	0.47	0.11	0.49	0.08	3.56 *
(iv) PTA efficiency	0.70	0.33	0.93	0.34	1.11	0.63	4.21 *
(iv) Triangle Completion	21.95	11.95	25.55	12.79	13.31	7.98	7.08 **

** *p* < 0.01, * *p* < 0.05.

**Table 3:** Pearson bivariate correlation between measures of various visual-spatial abilities.

Assessment	1	2	3	4
1. R-PDT Time 1	--			
2. Triangle completion task	0.22	--		
3. PTA efficiency	0.29 *	0.21	--	
4. MR efficiency	0.08	0.20	0.34 **	--

** Correlation is significant at the 0.01 level (two tailed). * Correlation is significant at the 0.05 level (two tailed).

**Table 4:** Examples of participants’ verbal reports.

Questions Asked by the Interviewer	Allocentric-Survey Mappers	Egocentric-Survey Mappers
What cues did you use to remember this route?	“I remember recalling the corridor first, and then from the starting point, I recall the relative position on one floor with respect to another.”	“I considered the distance between landmarks, sequence and location with respect to where I was.” “The sequence of landmarks and right and left turns. Used bigger landmarks for orientation and the general landscape as well.”
What do you remember from navigating the route?	“I remember it was like…not a straight corridor…it turned…it’s slanted. … I think it slanted to the…slanted right.” “We walked a little, and then we took a 45-degree turn to the left.” “I was basically making a round by the corner of the building so you go back so you turn right, turn right again so you pass by Sembcorp, which should be on top of the IBM then.” “Straight path and then made a 45-degree angled turn to the left, walked straight all the way to the endpoint.” “Long corridor, and then the bench was on the right side of it.”	“Facing the Sembcorp lab, we turned left. In front of me, on the right, was LT1 The staff lounge was on my right.” “Before we took the stairs, I could see the orange board, with words. After that, the orange board was like right in front… and then the staff lounge was on the right.” “When we came out of the stairs, I could see Sembcorp in front of me.” “From the link, you can see that the greenery that was there earlier and above, when we took the stairs, it became closer and at eye level”. “As we faced Cheers, we turned right… The unsheltered pathway was on my left. I saw LT1 on my right.”
How do you picture the route when you think about it? Do you imagine it from the top-down (bird’s-eye view) or first-person (as you were on the route) perspective?	“Definitely tried to create a top-down perspective. I was picturing myself on the route when I was navigating but not when I try to recall the route. I can’t picture this during recall.” “When I think about it, it looks the same to me as it would have on google maps.”	“No top-down perspective. I use a first-person perspective but only at landmarks. I mean I remember how the landmarks looked like from my viewpoint.”

**Table 5:** I-PDT trials with and without the SWM distractor task matched for the difficulty level.

IPDT without a Distractor			IPDT with a Distractor	
Trial	Imagined Heading	Pointing Direction	Imagined Heading	Pointing Direction
1	100°	BL	100°	BL
2	100°	BR	100°	FL
3	100°	BL	120°	BR
4	120°	BL	120°	FR
5	120°	FL	120°	BR
6	140°	BL	140°	BL
7	140°	FR	140°	FL
8	140°	FR	160°	FL
9	160°	FL	160°	BR
10	180°	BR	160°	FR
11	180°	FR	180°	BL
12	180°	BR	180°	FL

**Table 6:** Descriptive statistics and ANOVA results for I-PDT accuracy and RT without SWM task (I-PDT-11) and with SWM task (I-PDT 2) by type of sketch = map category.

Task	Procedural/Route Map Sketcherss		Allocentric-Survey Map Sketchers		Egocentric-Survey Map Sketchers		ANOVA Results
	Mean	SD	Mean	SD	Mean	SD	*F*(2, 44)
(i) I-PDT-1	0.30	0.18	0.51	0.18	0.53	0.24	5.69 **
(ii) I-PDT-2	0.35	0.16	0.50	0.14	0.56	0.22	5.65 **
(iii) I-PDT-1 RT (s)	14.80	6.28	12.86	2.91	10.95	2.28	1.47
(iv) I-PDT-2 RT (s)	15.20	5.48	16.53	3.85	11.94	2.60	4.59 *
(vi) MR—efficiency	0.36	0.08	0.44	0.04	0.40	0.06	7.04 **
(vii) PTA—minefficiency	0.59	0.21	0.66	0.14	0.81	0.31	3.49 *

** *p* < 0.01, * *p* < 0.05.

## Data Availability

The data presented in this study are available on request from the corresponding author. The data are not publicly available due to privacy reasons.
